# Cerebral tissue saturation, the next step in cardiopulmonary resuscitation management?

**DOI:** 10.1186/s13054-014-0583-0

**Published:** 2014-11-01

**Authors:** Cornelia Genbrugge, Willem Boer, Ingrid Meex, Frank Jans, Jo Dens, Cathy De Deyne

**Affiliations:** Faculty of Medicine and Life Sciences, Hasselt University, Martelarenlaan 42, Hasselt, 3500 Belgium; Department of Anesthesiology, Intensive Care, Emergency Medicine and Pain Therapy, Ziekenhuis Oost-Limburg Genk, Schiepse Bos 6, Genk, 3600 Belgium; Department of Cardiology, Ziekenhuis Oost-Limburg Genk, Schiepse Bos 6, Genk, 3600 Belgium

The goal of cardiopulmonary resuscitation (CPR) is to preserve the pre-arrest neurological state by maintaining sufficient cerebral blood flow and oxygenation, but the predictors thereof remain largely unknown. Despite recent attempts to improve the quality of basic and advanced life support, no monitored link to the neurological and physiological response of these CPR efforts has been established. The difficult decision to end pre-hospital resuscitation efforts is currently based on the circumstances of cardiac arrest, length of resuscitation efforts (if available), knowledge of pre-morbid physiological reserves, and (if present) end-tidal carbon dioxide (ETCO_2_) measurement. ETCO_2_ is currently the only parameter proven to correlate with the likelihood of return of spontaneous circulation (ROSC), although the prediction of long-term outcome based on ETCO_2_ values has not been established [1,2]. To measure ETCO_2_ adequately, invasive airway management is necessary and measured values are influenced by different lung pathologies.

By using two sensors on the forehead, near infrared spectroscopy (NIRS) measures the regional difference between oxygenated and deoxygenated hemoglobin, which expresses the difference in oxygen supply and demand. It determines cerebral tissue saturation non-invasively and independent of pulsatile flow. Müllner and colleagues [3] were the first to examine the use of cerebral oxygenation in (post-)cardiac arrest circumstances. They recorded cerebral saturation in six patients with ongoing CPR in the emergency department. Patients who achieved ROSC had higher cerebral saturation values compared with the single patient without ROSC. Cerebral NIRS was also studied during coronary artery bypass surgery. A correlation between desaturation and cognitive dysfunction [4], prolonged length of hospital stay [5], and perioperative cerebrovascular accident [6] was observed, and accordingly two landmark studies [7,8] showed that a goal-directed protocol preventing cerebral desaturation resulted in a decrease in length of intensive care unit and hospital stay, lower incidence of major organ morbidity and mortality, and decreased risk of cognitive decline [4-8].

Almost 20 years after the first published study on cerebral saturation monitoring during CPR, a revival of cerebral saturation measurement during CPR is taking place. Recent published research measures cerebral saturation in patients with ongoing CPR at arrival to the emergency department, but different cerebral saturation devices and different methods for analysis of NIRS data are used. The latest research on NIRS in the CPR setting focuses on two main questions.

Firstly, can cerebral saturation values predict ROSC or neurological outcome? Ito and colleagues [9] observed higher initial cerebral saturation values for patients with a good outcome at hospital discharge and 90 days after cardiac arrest. Likewise, Parnia and colleagues [10] observed a higher mean cerebral saturation in patients achieving ROSC compared with patients without ROSC. However, this study incorporated cerebral saturation values measured at the moment of ROSC into their mean values. This could have led to an overestimation of the mean cerebral saturation values in patients achieving ROSC. In a small study, patients with ROSC had a rise in cerebral saturation during CPR in contrast to patients without ROSC [11]. No patients without an increase in cerebral saturation achieved ROSC.

The second question recently addressed is whether cerebral saturation values can guide CPR efforts. Cerebral saturation measurements are probably more useful as a dynamic measurement instead of a static, single value. Hence, low cerebral saturation values could be interpreted as a need for interventions aimed at improving cerebral oxygenation during CPR. Sustained low cerebral saturation values, despite these interventions, could indicate futile CPR efforts. It has been noted that no patients with a mean cerebral saturation of less than 30% achieved ROSC [12]. In contrast, patients achieving ROSC spent the majority of time during CPR with saturation values of above 30% [10]. Furthermore, patients with ROSC showed a marked increase in cerebral oxygenation throughout advanced life support compared with non-survivors. As post-cardiac arrest patients are often hemodynamically unstable the first minutes after arrest and monitoring parameters are often unreliable during transport, NIRS could fulfill an important role in this setting. Meex and colleagues [13] noticed a speedy decline in cerebral saturation when re-arrest occurred during transport. These findings are similar to those described by Frisch and colleagues [14] (tissue saturation) and in case reports analyzing cerebral saturation during transcatheter aortic valve implantation [15,16].

In conclusion, preliminary data suggest that monitoring of cerebral saturation during CPR seems a likely predictor of both ROSC and neurological outcome and that it might have a role guiding CPR interventions. Although the current knowledge, obtained from small observational studies, is limited, both the further development of NIRS devices and the likely execution of well-designed large blinded observational trials, particularly in the difficult environment of out-of-hospital CPR, bode well for the future. A real-time monitoring tool providing vital information on the neurological and physiological response to CPR efforts and with predictive value for neurological outcome seems close at hand.

## Abbreviations

CPR: Cardiopulmonary resuscitation
ETCO_2_: End-tidal carbon dioxide
NIRS: Near infrared spectroscopy
ROSC: Return of spontaneous circulation
